# METTL5 serves as a diagnostic and prognostic biomarker in hepatocellular carcinoma by influencing the immune microenvironment

**DOI:** 10.1038/s41598-023-37807-5

**Published:** 2023-07-03

**Authors:** Lei Wang, Jin-lin Peng

**Affiliations:** 1 Department of Rehabilitation Medicine, Hunan Provincial People’s Hospital, The First Affiliated Hospital of Hunan Normal University, Changsha, 410001 Hunan Province China; 2 Department of Gastroenterology, Tongji Hospital, Tongji Medical College, Huazhong University of Science & Technology, Wuhan, 430030 Hubei Province China

**Keywords:** Tumour biomarkers, Tumour immunology, Liver cancer

## Abstract

Despite the abnormal expression of 18S rRNA m6A methyltransferase METTL5 being reported in some types of human malignancies, but its effect on hepatocellular carcinoma (HCC) remains to be unclear. This study aims to elucidate the influences of METTL5 on the carcinogenesis and progression of HCC. Expressions of METTL5 gene, transcript, protein, and promoter methylation in HCC were examined through multiple databases, c-BioPortal was used to confirm the genomic alterations of METTL5, the biological functions, target networks of kinases and microRNAs of METTL5, and its interactive differential genes were investigated through LinkedOmics. The possible correlation of METTL5 with the tumor-related infiltration of immune cells for HCC were explored comprehensively by using the online tools of TIMER and TISIDB. Expressions of METTL5 gene, mRNA, and protein were considerably overexpressed in HCC samples in comparison with healthy samples. The high methylation of the METTL5 promoter was observed in HCC tissues. Elevated METTL5 expression exhibited unfavorable survival outcomes in HCC patients. METTL5 expression were enriched in the signaling pathways of ribosome and oxidative phosphorylation, mismatch repair, and spliceosome through the involvement of several cancer-related kinases and miRNAs. The METTL5 expression has a positive correlation with the infiltration degree of B cells, CD8+ T cells, CD4+ T cells, macrophages, neutrophils, and dendritic cells in HCC. Marker genes of tumor immune-infiltrated cells have strong connection with METTL5. Furthermore, the upregulation of METTL5 was strongly correlated with the immune regulation of immunomodulators, chemokines, and chemokine receptors in the immune microenvironment. The oncogenesis and development of HCC are closely related to METTL5 expression, and the overexpression of METTL5 resulted in the poor survival outcome of HCC patients by regulating tumor immune microenvironment.

## Introduction

Liver cancer remains a global threat to the physical and mental health of humans and is characterized by high mortality and great incidence^1^. Hepatocellular carcinoma (HCC or LIHC) is among the most prominent subtypes of all primary intrahepatic malignancies, encompassing approximately 90% of all liver cancer cases. HCC was the sixth most prevalent malignancy in terms of morbidity and is considered the third most frequent cause in terms of malignancy related mortality all over the world^2^. The diagnoses of HCC were identified on the basis of radiologic image, serologic molecular markers, or pathologic findings. However, a large proportion of HCC cases from the initial diagnosis involved terminal stage cases accompanied by high mortality. Despite the rapid advances in the effective treatment strategies of hepatectomy, chemotherapy, radiotherapy, immunotherapy, and targeted therapy, the survival outcome of HCC patients remains unsatisfactory because of its highly invasive nature^3^. The five-year survival rate of a small percentage of HCC-diagnosed population was expected to exceed 15% because of its easy recurrence and metastasis^4^. Thus, the potential diagnostic and prognostic indexes of HCC are urgently needed to provide new therapeutic HCC targets.

Ribosomal RNA (rRNA) comprised approximately 80% of the total cellular RNA in eukaryotes. rRNA modification emerged as a momentous post-transcriptional gene regulation process. The N6 methyladenosine (m6A) modification that occurred on rRNA influenced the modulation of ribosome structure and function^5,6^. Methyltransferase N6-adenosine (METTL5) served as a rRNA m6A and was recently identified to specifically catalyze human 18S rRNA N6 methylation at adenosine 1832 site^7^, which possessed vital importance in regulating the function and development of ribosomes^7,8^.

Abnormal METTL5 expression was observed in numerous human malignancy types. METTL5 expression was highly upregulated in lung adenocarcinoma and was bound up with short survival time^7,9^. METTL5 overexpression promoted translation initiation and cell growth in breast cancer^7,8^. The effects of METTL5 on the tumorigenesis and the development of HCC remains to be rarely investigated.

The expression characteristics of METTL5 in HCC samples were further explained in this research. We aim to evaluate the underlying molecular mechanisms responsible for HCC onset and the potential role of METTL5 expression on HCC prognosis.

## Results

### Expression levels of METTL5 gene in HCC

**Figure 1:**
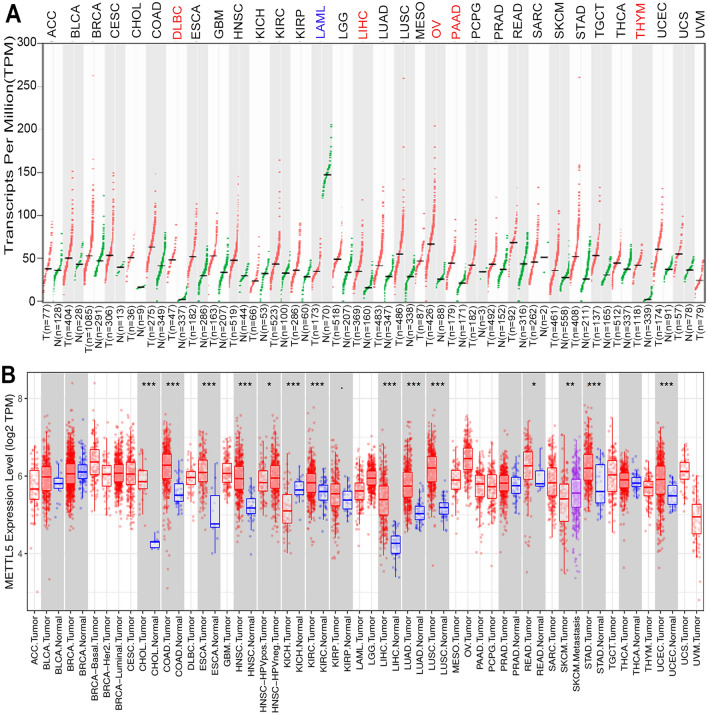
Expression levels of METTL5 gene in Pan-cancer. (**A**) Expression profiles of the METTL5 gene in different cancer types and paired normal tissue samples from the GEPIA database. (**B**) Expression profiles of the METTL5 gene in different cancer types and paired normal tissue samples from the TIMER database.

**Figure 2:**
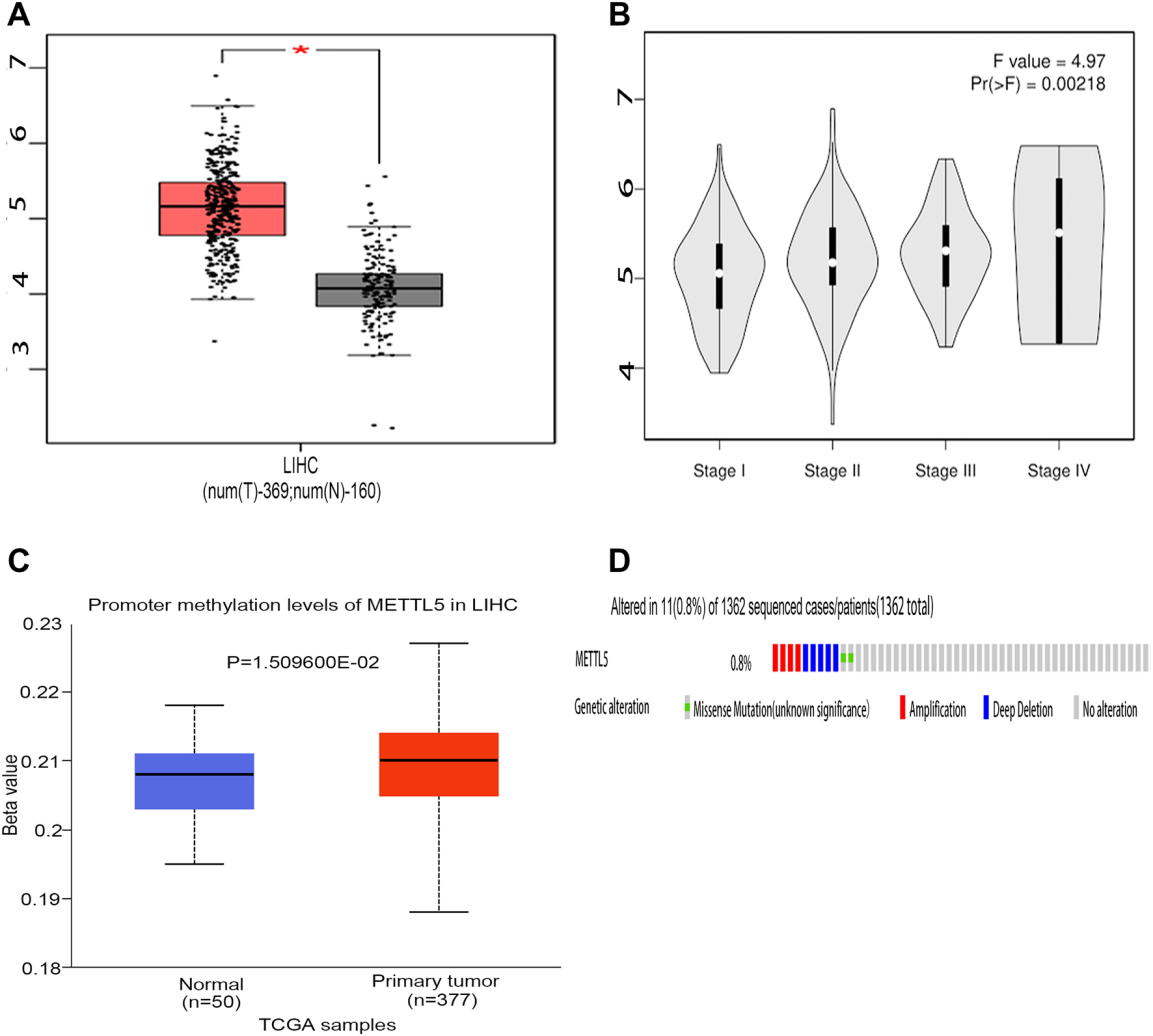
Expression profiles of the METTL5 gene in hepatocellular carcinoma (LIHC). (**A**) Expression profiles of the METTL5 gene in LIHC and paired of normal tissues from the GEPIA database. (**B**) Correlation between expression of METTL5 gene and Cancer stage of LIHC through the GEPIA database. (**C**) Promoter methylation levels of METTL5 in LIHC were evaluated by box plot using the UALCAN database. (**D**) METTL5 alterations in LIHC through the cBioPortal database.

We calculated the differential expression of the METTL5 gene in various types of cancers and matched normal tissues by means of online searching at the GEPIA database (Fig. 1A). The METTL5 gene was markably elevated among HCC specimens in comparison with surrounding healthy tissues (Fig. 2A). Further analyses were adopted for assessing METTL5 gene expression in relation to tumor stage, and the METTL5 gene expression gradually increased among the advent of HCC progression via GEPIA (Fig. 2B). Such finding was consistent with the outcomes obtained from the Timer database (Fig. 1B). The aforementioned data implied that METTL5 gene overexpression may play a significant role in the tumorigenesis for HCC.

### Potential prognostic effects of METTL5 gene expression on HCC

**Figure 3:**
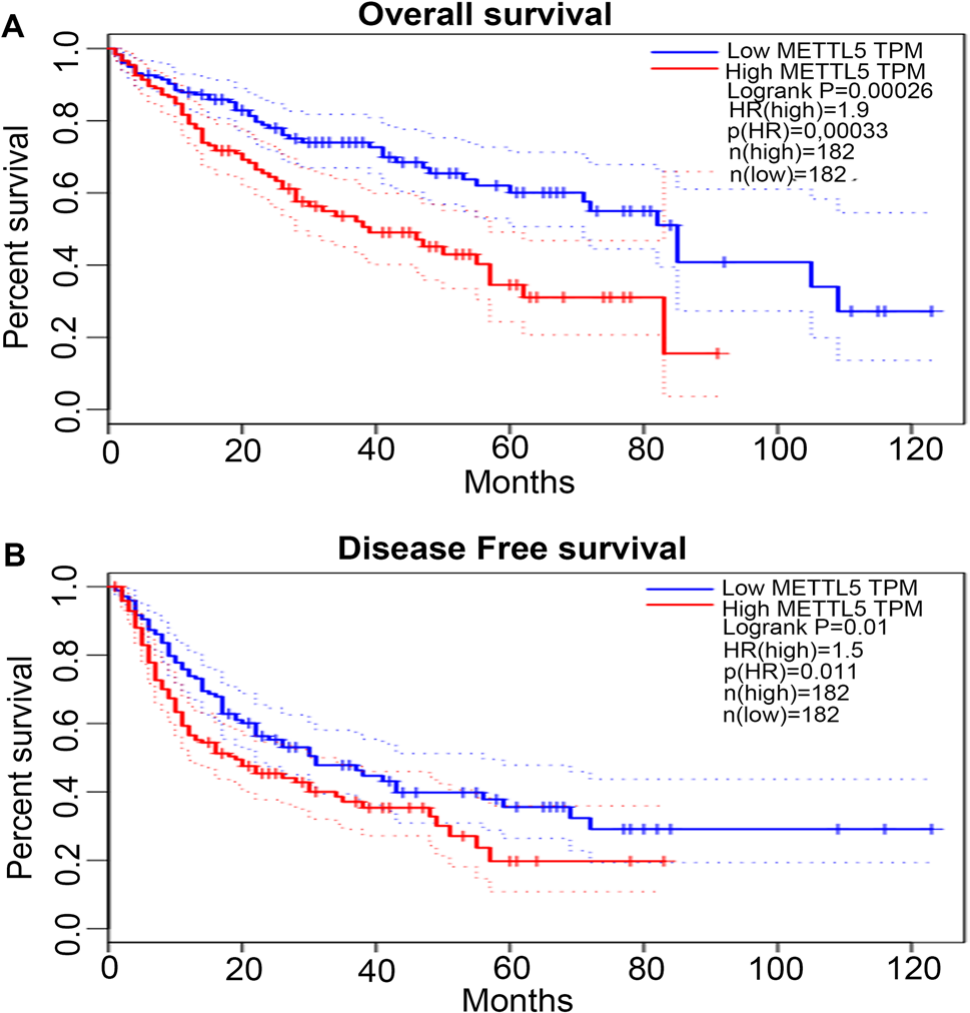
Comparisons of the effects of high and low expression levels of METTL5 gene on survival time of hepatocellular carcinoma (LIHC) patients using GEPIA database. (**A**) and (**B**) Revealed that elevated expression levels of METTL5 gene were associated with worse overall survival and disease free survival for LIHC patients.

We evaluated the prognostic significance of METTL5 expression on HCC patients using GEPIA. METTL5 gene overexpression is disadvantageous in terms of the overall survival time (OS) (Fig. 3A) and disease-free survival (DFS) (Fig. 3B) of HCC patients.

### Expression profiles of METTL5 transcript in HCC

**Figure 4:**
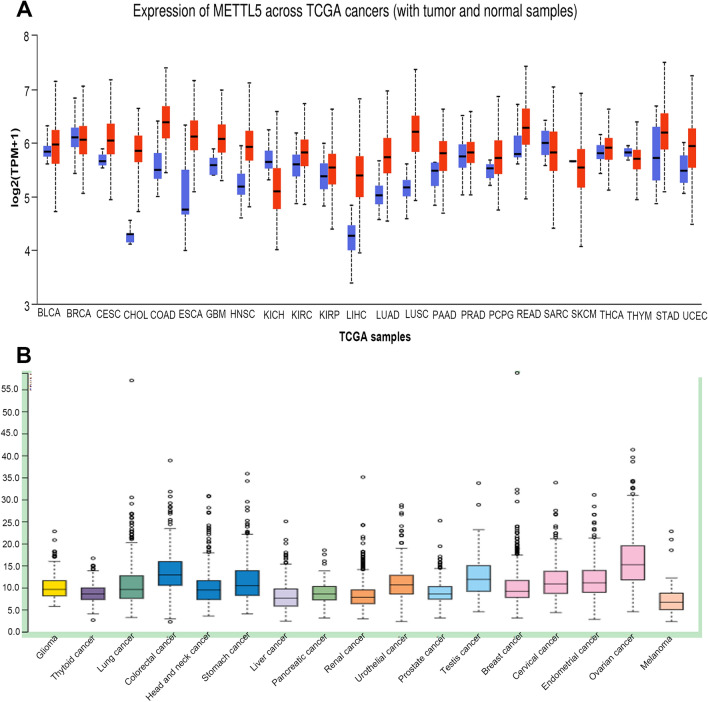
Expression profiles of the METTL5 transcript in hepatocellular carcinoma (LIHC): (**A**) expression profiles of the METTL5 transcript in different cancer types and paired normal tissue samples from the UALCAN database. (**B**) Expression profiles of the METTL5 gene in different cancer types from the HPA database.

**Figure 5:**
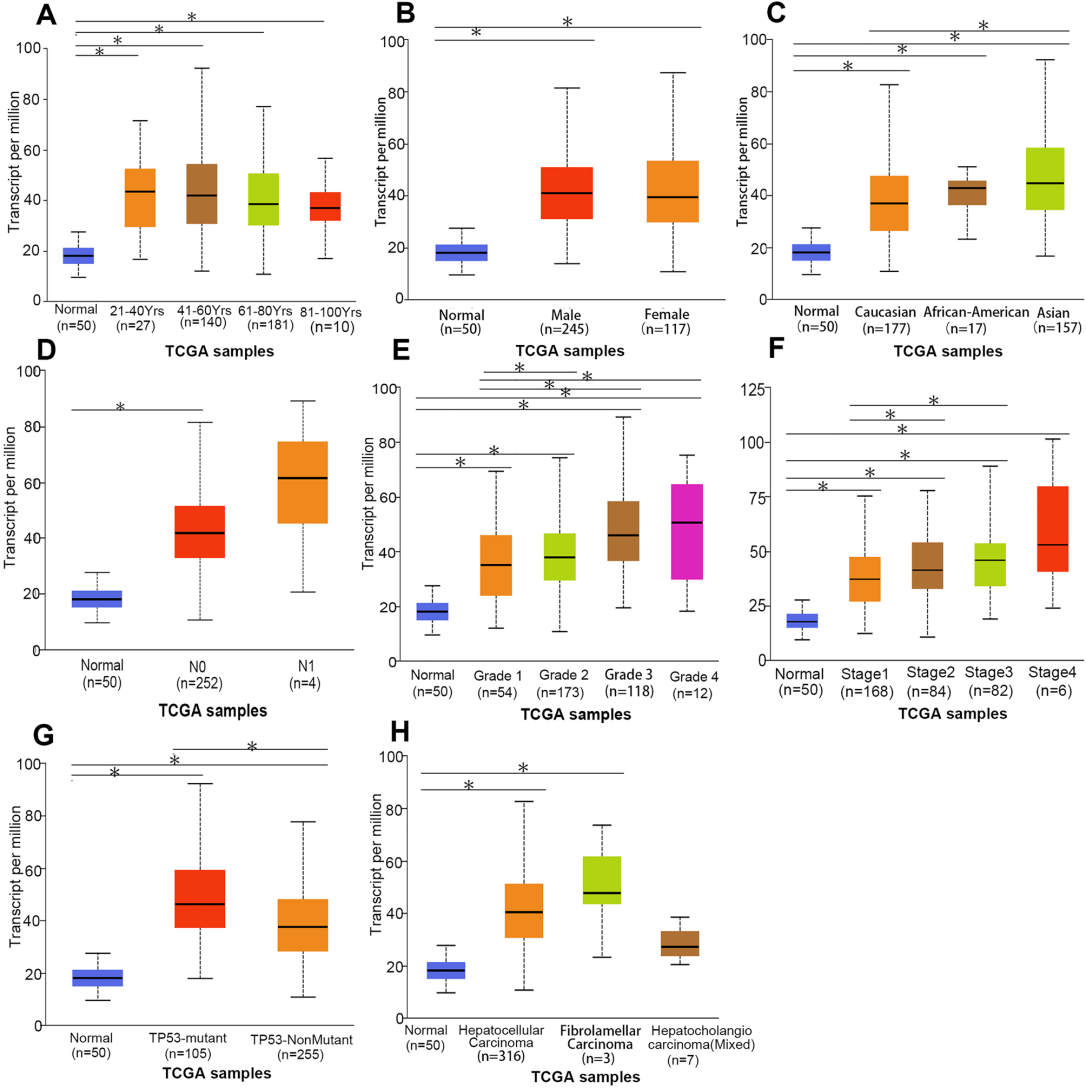
Expression of METTL5 transcription in different clinical characteristics of patients with liver hepatocellular carcinoma (LIHC) via UALCAN. (**A**) Correlation of METTL5 transcription with different patients age. (**B**) Correlation of METTL5 transcription with different patients sex. (**C**) Correlation of METTL5 transcription with different patients race. (**D**) Correlation of METTL5 transcription with different nodal metastasis status. (**E**) Correlation of METTL5 transcription with different cancer grade. (**F**) Correlation of METTL5 transcription with different cancer stage. (**G**) Correlation of METTL5 transcription with different TP53 mutation Status. (**I**) Correlation of METTL5 transcription with different histological subtypes.

The available expression data of the METTL5 transcript in multiple HCC tissues were evaluated online from the UALCAN and HPA databases. Findings from both databases revealed that the intensified mRNA expression of METTL5 was observed in HCC specimens in comparison with adjacent healthy tissues via UALCAN (Fig. 4A) which is consistent with the outcomes of HPA database(Fig. 4B). Further analyses were used to assess the METTL5 transcript expression in relation to various clinical characteristics of HCC by using UALCAN. The results proved that METTL5 mRNA in HCC specimens was up-regulated in terms of age from 21 to 40, N1, stage 4, grade 4, Asian race, TP53 mutation, and male patients (Fig. 5).

### Potential prognostic effect of METTL5 transcript expression on HCC

**Figure 6:**
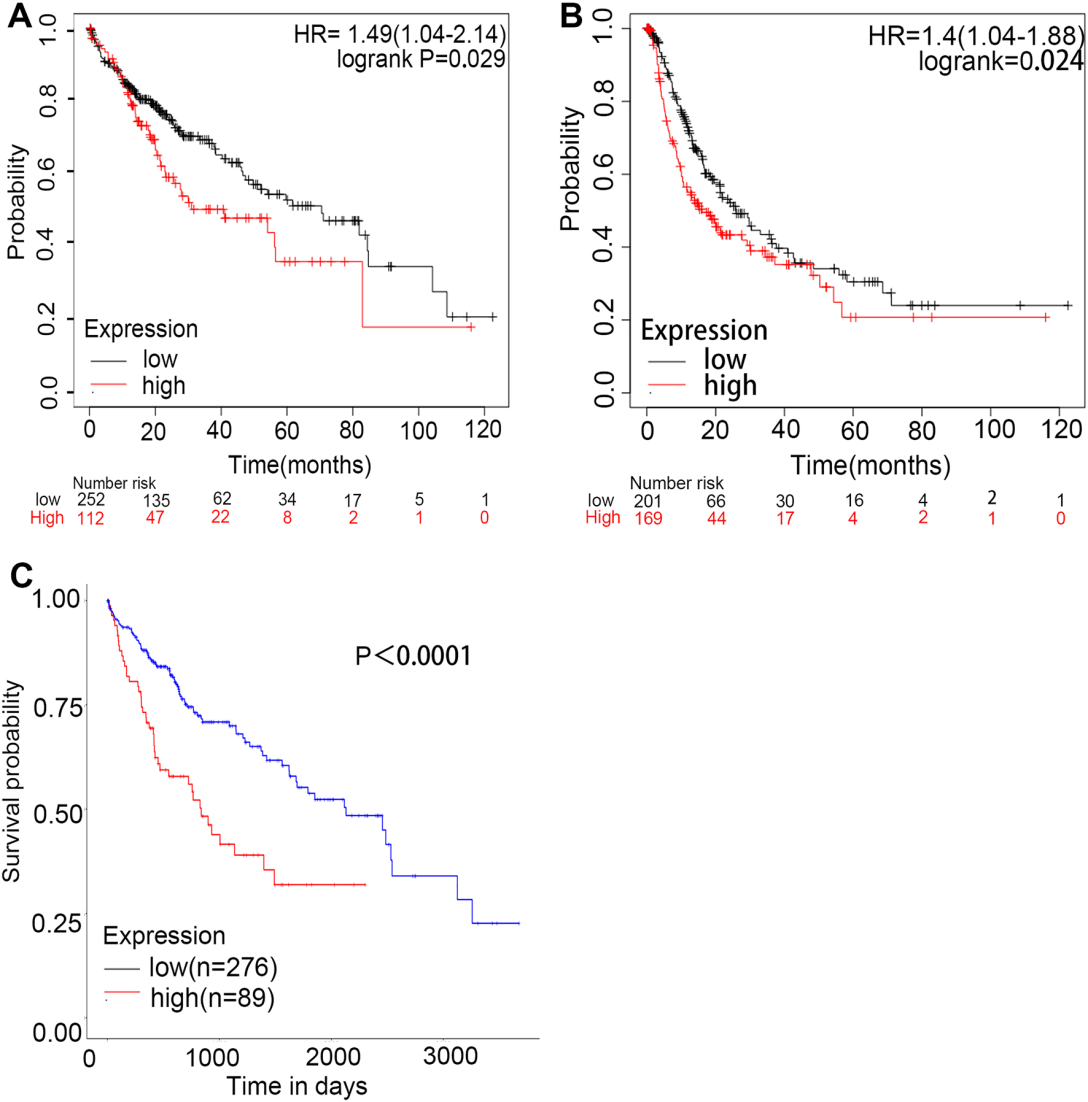
Comparisons of the effects of high and low expression levels of METTL5 transcript on survival time of hepatocellular carcinoma (LIHC) patients. (**A**) and (**B**) Elevated expression levels of METTL5 transcript were associated with worse overall survival and progression free survival for LIHC patients using K-M plotter. (**C**) Effect of METTL5 transcript expression on LIHC patient survival via UALCAN.

We further evaluated the impact of METTL5 mRNA expression on the survival outcomes of HCC patients using the KM database. The results showed that METTL5 mRNA overexpression had a close association with poor OS and PFS in HCC patients (Fig. 6A and B). We also confirmed that increased METTL5 mRNA expression was strongly linked with unfavorable OS and PFS in stage 3, stage 3 + 4, AJCC T3, drinking alcohol hobby, no infection with Hepatitis virus diseases, and male HCC patients (Table 1). The substantially increased expression of METTL5 transcript had link with disadvantageous survival via UALCAN (Fig. 6C). These data implied that the overexpression of METTL5 mRNA in HCC predicted poor prognostic outcomes.

**Table 1:** Correlation of METTL5 mRNA expression with Overall survival and progression-free survival in hepatocellular carcinoma with different clinicopathological features via KM plotter database.

Clinicopathological parameters	N	Overall survival(364)		Progression-free survival	
		Hazard ratio	logrank P	Hazard ratio	logrank P
Stage					
1	171	1.67 (0.9 − 3.11)	0.1	0.74 (0.44 − 1.23)	0.24
2	86	0.66 (0.3–1.47)	0.31	1.38 (0.76–2.52)	0.29
3	85	2.44 (1.32–4.49)	**0.0031**	2.01 (1.17–3.48)	**0.01**
4	5	–	**–**	–	–
1 + 2	257	1.46 (0.89–2.38)	0.13	1.25 (0.85–1.82)	0.25
3 + 4	90	2.43 (1.35–4.37)	**0.0022**	1.96 (1.15–3.34)	**0.012**
AJCC_T					
T1	181	1.55 (0.85–2.83)	0.15	0.78 (0.47–1.27)	0.31
T2	94	1.37 (0.62–3.06)	0.44	1.4 (0.74–2.63)	0.3
T3	80	2.47 (1.33–4.59)	**0.0032**	1.98 (1.13–3.5)	**0.016**
T4	13	–	–	–	**–**
Vascular invasion					
No	205	0.64 (0.38–1.07)	0.087	0.65 (0.42–1.01)	0.056
micro	93	0.58 (0.26–1.28)	0.17	1.64 (0.9–2.98)	0.1
Gender					
Male	250	1.9 (1.22–2.97)	**0.0041**	1.57 (1.09–2.26)	**0.015**
Female	121	0.73 (0.42–1.29)	0.28	1.25 (0.74–2.11)	0.41
Alcohol consumption					
No	205	1.49 (0.93–2.37)	0.095	1.38 (0.92–2.06)	0.12
Yes	117	1.96 (1–3.82)	**0.046**	1.9 (1.02–3.54)	**0.041**
Hepatitis virus					
No	169	1.9 (1.19–3.04)	**0.0061**	1.75 (1.13–2.72)	**0.012**
Yes	153	1.56 (0.81–3)	0.18	0.72 (0.44–1.18)	0.19

Significant values are in [bold].

### Expression profiles of METTL5 protein in HCC patients

**Figure 7:**
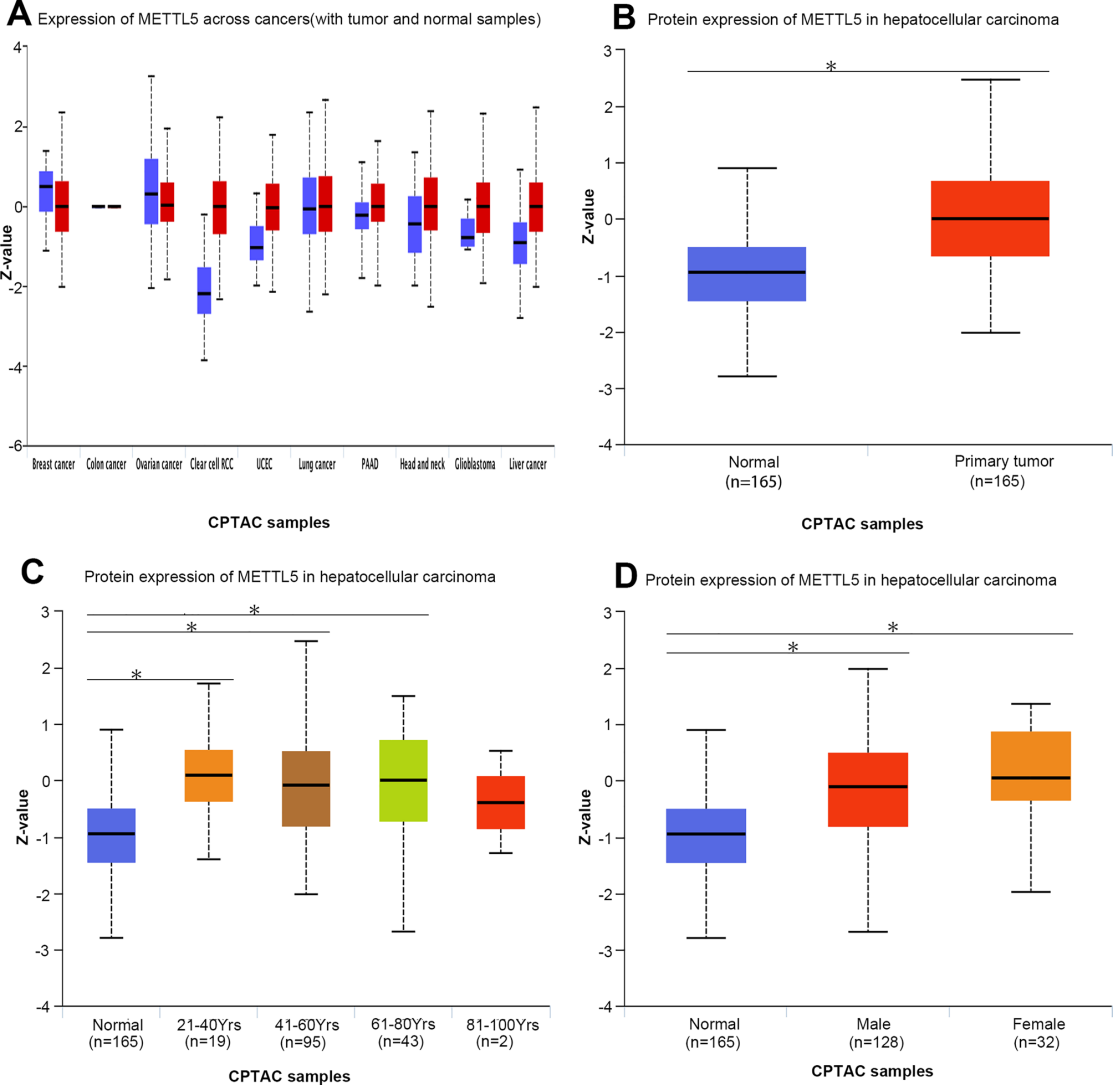
METTL5 transcription in subgroups of patients with hepatocellular carcinoma via UALCAN. (**A**) Boxplot showing relative expression of METTL5 transcription in pancancer samples. (**B**) Boxplot showing relative expression of METTL5 transcription in normal and LIHC samples. (**C**) Boxplot showing relative expression of METTL5 transcription in normal individuals with different age in LIHC patients. (**D**) Boxplot showing relative expression of METTL5 transcription in normal individuals of gender in LIHC patients.

METTL5 protein expression was substantially elevated in liver cancer when compared with paired normal tissues through the UALCAN database (Fig. 7A and B). The upregulation of METTL5 protein was commonly detected in 21–40 year-olds and female patients via UALCAN (Fig. 7C and D).

### Potential prognostic effects of METTL5 protein expression on HCC patients

**Figure 8:**
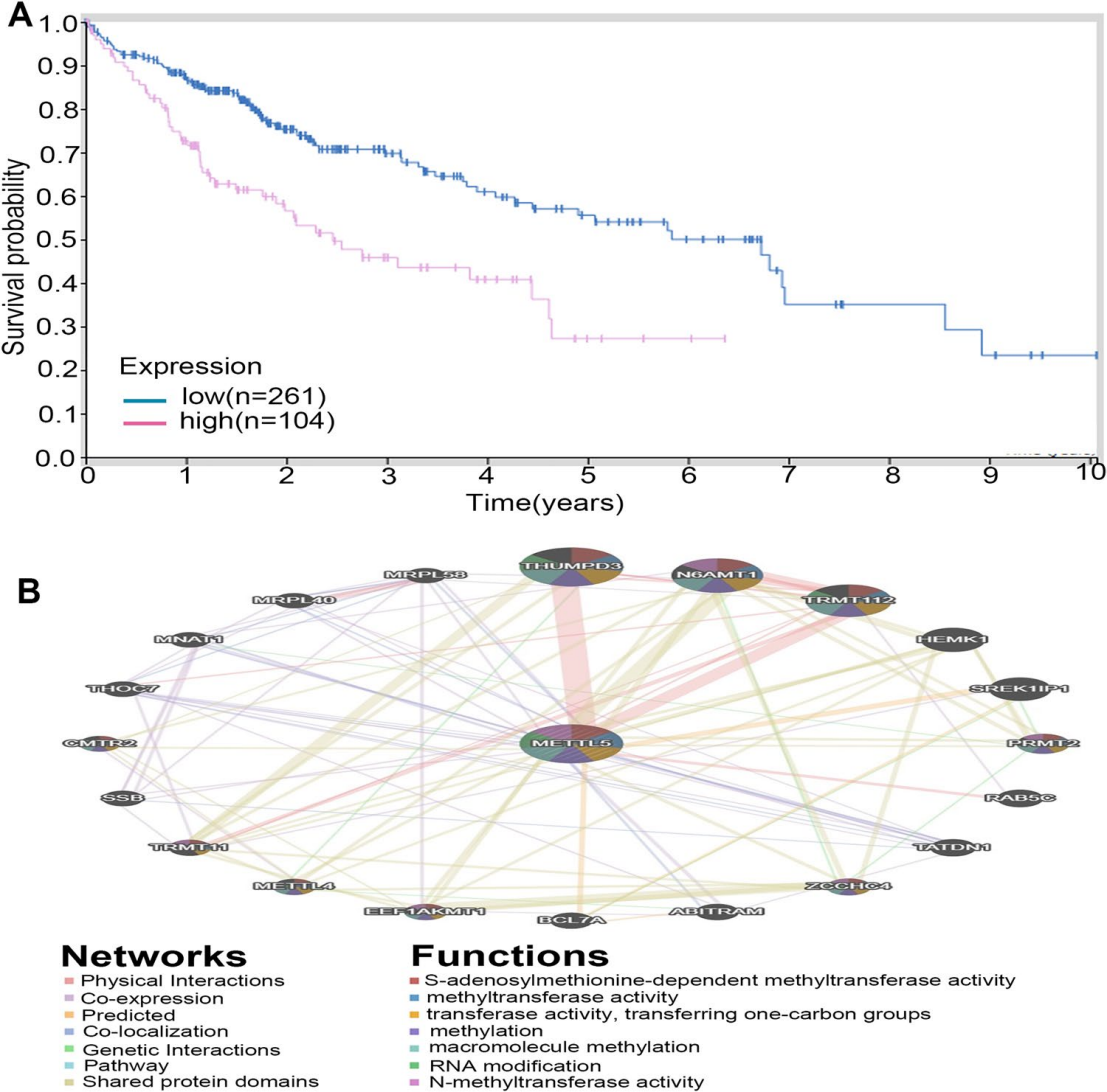
The prognostic value and biological function of METTL5 protein in LIHC samples. (**A**) Comparisons of the effects of high and low expression levels of METTL5 protein on survival time of hepatocellular carcinoma(LIHC) patients using HPA database. (**B**) Protein–protein interaction network of METTL5 was constructed by GeneMANIA.

We further evaluate the impact of METTL5 protein expression on the survival outcomes of HCC patients using the HPA database. The high expression of METTL5 protein generally indicated the poor survival outcomes of HCC patients (Fig. 8A).

### Genetic alteration of METTL5 in HCC patients

Hypermethylation of METTL5 gene was displayed in HCC in comparison with paired healthy specimens (Fig. 2C), inferring that the upregulation of METTL5 methylation contributed to METTL5 overexpression in HCC. Genetic alterations of METTL5 in HCC patients were analyzed using the cBioPortal tools. Our findings revealed that the genomic alterations of METTL5 occurred in 0.7% of HCC patients (Fig. 2D). All these results revealed that METTL5 genomic alteration indeed occur in HCC patients.

### Gene enrichment analysis of METTL5 in HCC patients

**Figure 9:**
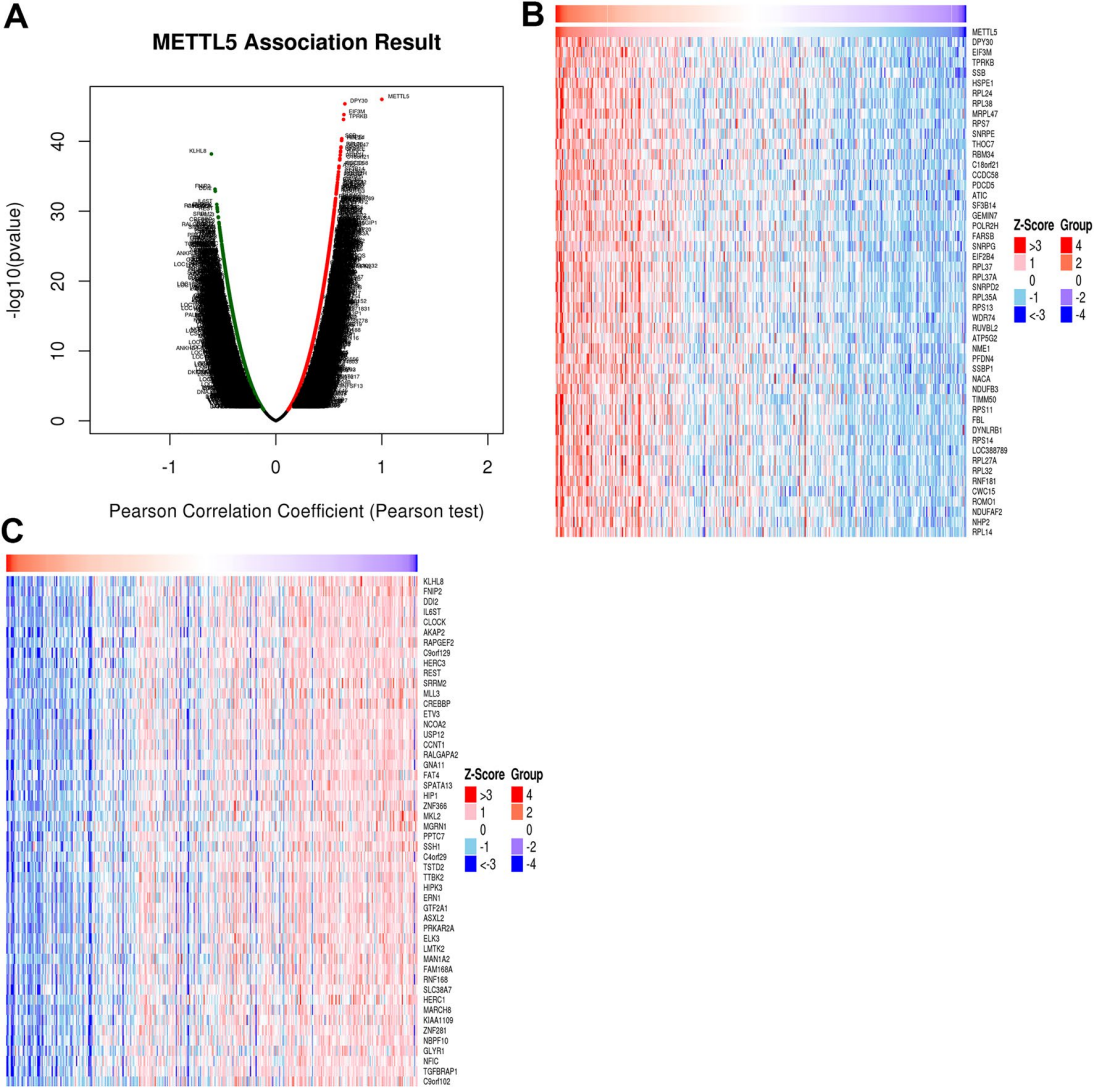
Genes differentially expressed in correlation with METTL5 in hepatocellular carcinoma via LinkedOmics. (**A**) A Pearson test was used to analyze correlations between METTL5 and genes differentially expressed in LIHC. (**B**) Heat maps showing the top 50 genes positively correlated with METTL5 in LIHC. (**C**) Heat maps showing the top 50 genes negatively correlated with METTL5 in LIHC.

**Figure 10:**
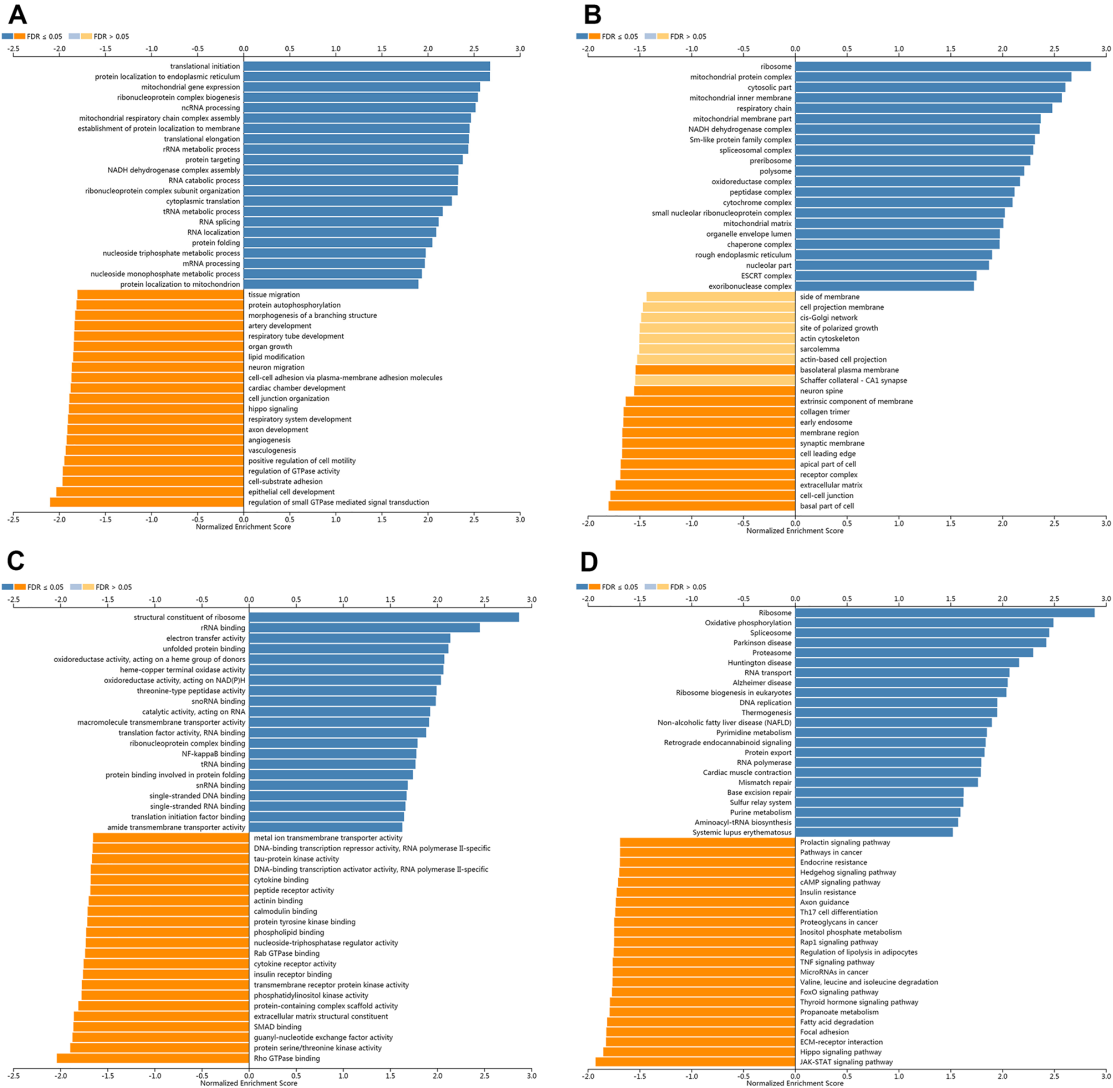
The GO and KEGG pathway analyses of METTL5 in LIHC samples. (**A**) Biological processes involved in METTL5 in LIHC. (**B**) Cellular components involved in METTL5 in LIHC. (**C**) Molecular functions involved in METTL5 in LIHC. (**D**) KEGG pathways involved in METTL5 in LIHC.

A total of 8600 genes correlated with METTL5 were shown in the volcano plot via LinkedOmics (Fig. 9A), which comprised 3568 positively correlative genes and 5032 adversely correlative genes. The top 50 most prominent genes with positive and negative connections with METTL5 were displayed (Fig. 9B and C). The METTL5-related enriched GO term and KEGG pathway were analyzed. Significant biological processes (BPs) indicated that METTL5-related genes were intimately engaged in translational initiation, protein localization to endoplasmic reticulum, mitochondrial gene expression, ribonucleoprotein complex biogenesis, and ncRNA processing (Fig. 10A). These interactive genes served as cellular components of ribosome, mitochondrial protein complex, cytosolic part, mitochondrial inner membrane, and respiratory chain (Fig. 10B). The molecular function enrichment of METTL5-related genes is mainly projected in ribosome, rRNA binding, electron transfer activity, unfolded protein binding, and oxidoreductase activity. Acting on a Heme group of donors with Heme-copper terminal oxidase activity (Fig. 10C), METTL5 were proven to largely participate in the signaling pathways of ribosome, oxidative phosphorylation, spliceosome, Parkinson disease, proteasome, and Huntington disease (Fig. 10D).

### Co-expressed protein correlated with METTL5

We further evaluated the major protein that interacted with METTL5 through the Metascape database. The results showed 20 types of METTL5 interactive proteins.

### PPI network of METTL5

The PPI network was used to visualize the interaction between METTL5 and its relevant protein through the use of GeneMANIA tools. METTL5 was mainly enriched in the regulation of S-adenosylmethionine-dependent methyltransferase activity, methyltransferase activity, transferase activity, transferring one-carbon groups, methylation, RNA modification, and N-methyltransferase activity (Fig. 8B).

### METTL5 networks of kinase and microRNA (miRNA) in HCC

**Figure 11:**
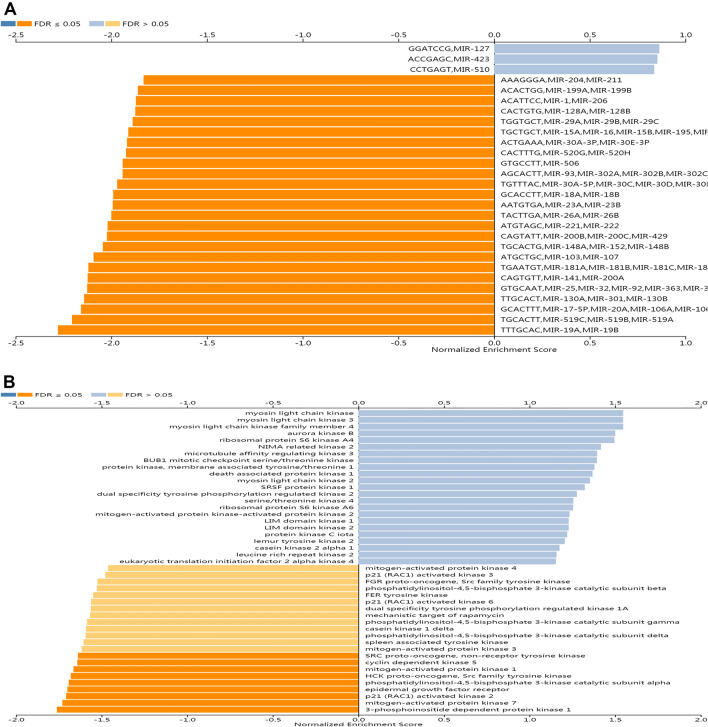
The Kinase and miRNA networks of METTL5 in hepatocellular carcinoma via LinkedOmics. (**A**) The miRNA networks of METTL5 in in hepatocellular carcinoma via LinkedOmics. (**B**) The Kinase networks of METTL5 in in hepatocellular carcinoma via LinkedOmics.

The top 5 most remarkable kinase-targets of METTL5 were primarily involved in the myosin light chain kinase, myosin light chain kinase3, myosin light chain kinase family member 4, aurora kinase B, and ribosomal protein S6 kinase A4 (Fig. 11B). The miRNA-target network was positively relevant to MIR-127, MIR-423, and MIR-510 (Fig. 11A).

### Correlation between METTL5 expression and immune biomarker in HCC

**Figure 12:**
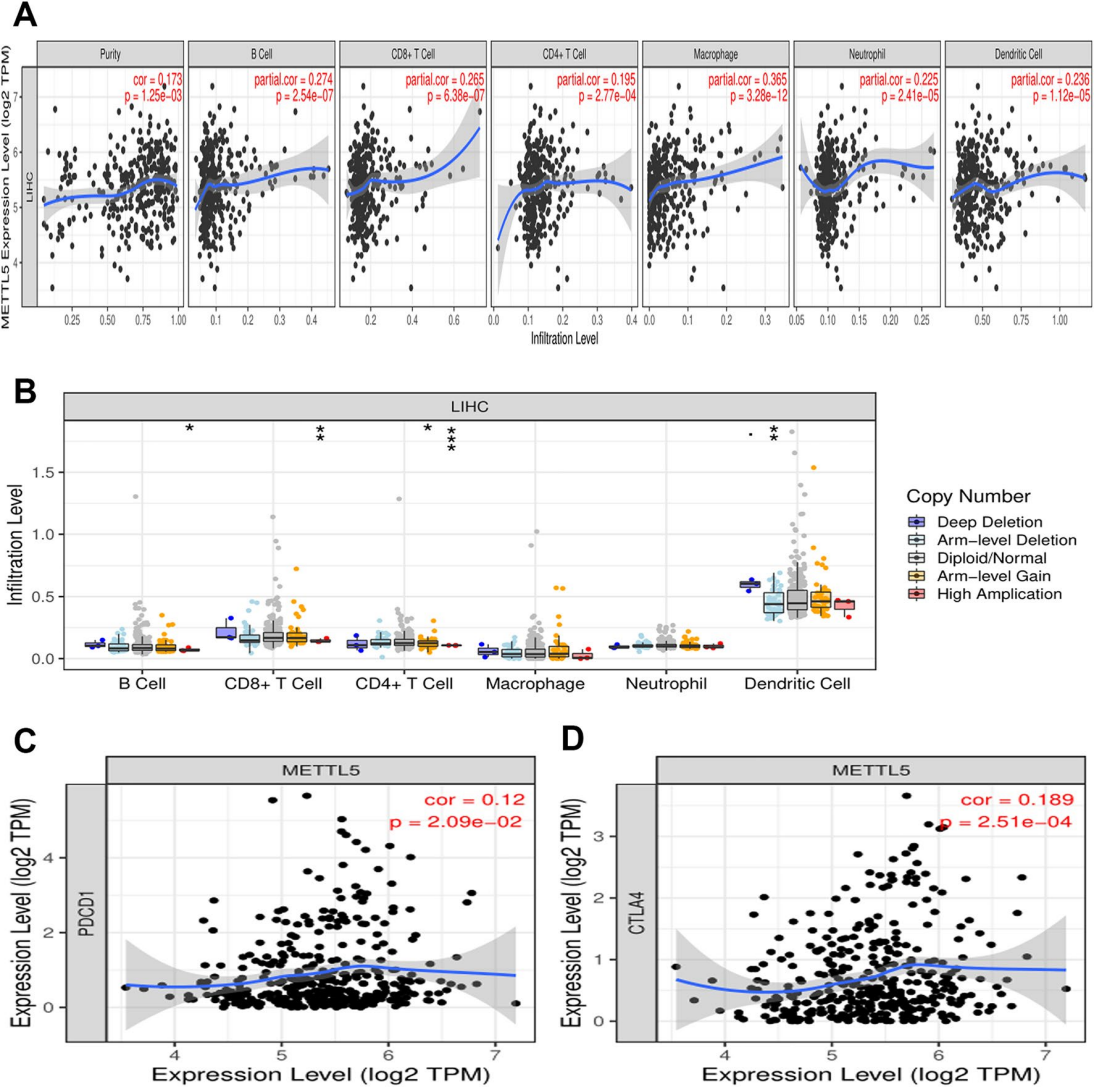
Correlation of METTL5 expression with immune infiltration in hepatocellular carcinoma(LIHC). (**A**) Correlation between the expression of METTL5 and the abundance of immune infiltration in LIHC at Timer database. (**B**) The infiltration level of various immune cells under different copy numbers of METTL5 in LIHC. (**C**) Correlation of METTL5 expression with immune checkpoint inhibitors.

Immune infiltration had a significant impact on tumor progression. No apparent change of immune cell infiltration under different copy numbers of METTL5 existed in HCC specimens (Fig. 12B). The TIMER database was used to comprehensively evaluate the correlation of METTL5 expression to immune cell infiltration in HCC. METTL5 expression showed positive correlation with B cells, CD8+ T cells, CD4+ T cells, macrophages, neutrophils, and dendritic cell infiltration levels in HCC (Fig. 12A). The cox regression analysis was established to estimate the effects of METTL5 and immune cell infiltration on the survival outcomes of HCC patients. The results indicated that the infiltrating degree of B cells (coef = − 8.430, p = 0.017) and CD8+ T cells (coef = − 6.787, p = 0.008) had a positive correlation with the survivorship risk of HCC patients. Dendritic cells (coef = 5.563, p = 0.002) and METTL5 (coef = 0.562, p = 0.001) were available as independent risk factors of survival in HCC cases, whereas CD4+ Tcell (p = 0.159), macrophage (p = 0.106), and neutrophils (p = 0.804) had no impact on the survival of HCC patients. METTL5 expression also had a positive link with PD1 (PDCD1) and CTLA4 in HCC based on the TIMER and GEPIA databases (Fig. 12C and D). The expression levels of METTL5 had a strong connection with 16 gene markers of immune cell in HCC samples (Table 2).

**Table 2:** Correlation analysis between METTL5 and biomarkers of immune cells in HCC determined by GEPIA database.

Immune cell	Gene biomarkers	R value	p value
B cell	CD19	0.12	**0.023**
	CD79A	0.063	0.22
CD8+ T cell	CD8A	0.095	0.067
	CD8B	0.14	**0.0092**
CD4+ T cell	CD4	0.0011	0.98
M1 macrophage	NOS2	− 0.036	0.49
	IRF5	0.23	**6.8e−06**
	PTGS2	− 0.012	0.82
M2 macrophage	CD163	0.076	0.15
	VSIG4	0.077	0.14
	MS4A4A	0.087	0.095
Neutrophil	CEACAM8	− 0.0069	0.9
	ITGAM	0.13	**0.016**
	CCR7	0.038	0.47
Dendritic cell	HLA-DPB1	0.09	0.085
	HLA-DQB1	0.066	0.2
	HLA-DRA	0.09	0.086
	HLA-DPA1	0.059	0.26
	CD1C	0.053	0.31
	NRP1	0.22	**3e−05**
	ITGAX	0.1	**0.047**
T cell (general)	CD3D	0.15	**0.0048**
	CD3E	0.068	0.19
	CD2	0.09	0.083
Monocyte	CD86	0.18	**0.00055**
	CSF1R	0.081	0.12
Th1	TBX21	0.021	0.68
	STAT4	0.085	0.1
	STAT1	0.21	**4.2e−05**
	IFNG	0.11	**0.032**
	TNF	0.011	0.84
	IL12A	0.29	**8.1e−09**
	IL12B	0.025	0.63
Th2	GATA3	0.11	**0.043**
	STAT6	0.072	0.17
	STAT5A	0.042	0.42
	IL13	0.045	0.39
Th17	STAT3	0.0061	0.91
	IL17A	− 0.035	0.5
Treg	FOXP3	− 0.14	**0.0058**
	CCR8	0.051	0.33
	STAT5B	0.052	0.32
	TGFB1	0.25	**7.6e−07**
	SPI1	0.13	**0.014**
Th22	CCR10	0.16	**0.0019**
	AHR	0.093	0.074

Significant values are in [bold].

### METTL5 expression associated with immunomodulators in HCC

**Figure 13:**
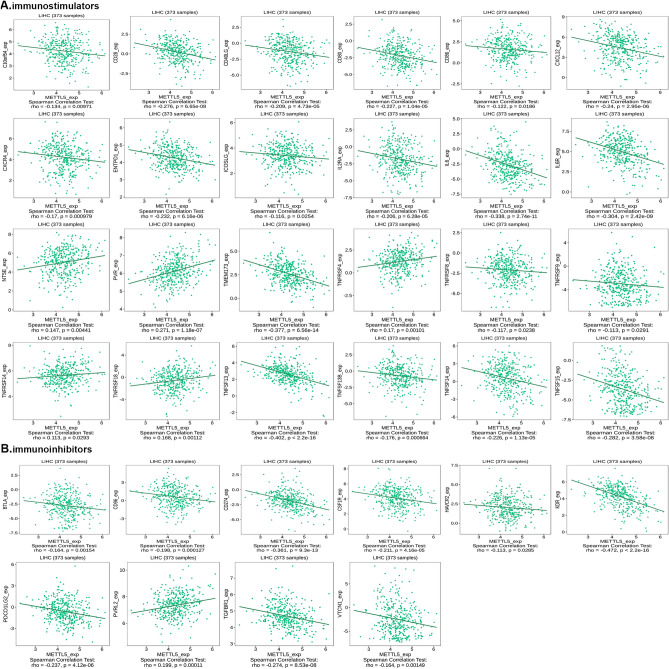
The expression of METTL5 is associated with immunomodulators in hepatocellular carcinoma. (**A**) Correlation between METTL5 expression and immunostimulators in hepatocellular carcinoma at TISIDB database. (**B**) Correlation between METTL5 expression and immunoinhibitors in hepatocellular carcinoma at TISIDB database.

TISIDB database was used to explore the possible correlation between METTL5 and various immune signatures. METTL5 expression had a positive correlation with the several immunostimulators of NT5E, PVR, TNFRSF4, TNFRSF14, and TNFRSF18 (Fig. 13A). Findings exhibited that METTL5 expression was negatively correlated with a large number of immunoinhibitors of BTLA, CD96, CD274, CSF1R, HAVCR2, KDR, PDCD1LG2, TGFBR1, and VTCN1 (Fig. 13B). The aforementioned results demonstrated the function of METTL5 as an immunoregulatory factor for HCC.

### Correlation between METTL5 expression and chemokines in HCC

**Figure 14:**
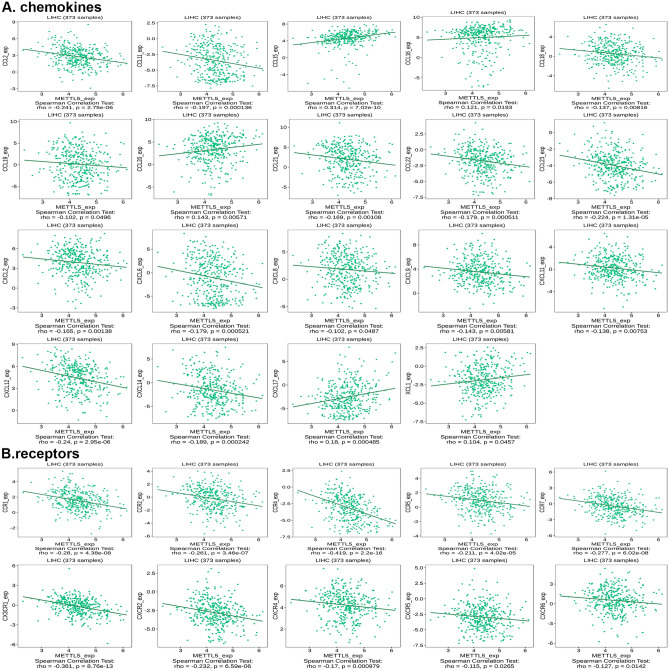
Correlation between the expression of METTL5 and chemokines in hepatocellular carcinoma. (**A**) Correlation between METTL5 expression and chemokines in hepatocellular carcinoma at TISIDB database. (**B**) Correlation between METTL5 expression and chemokine receptors in hepatocellular carcinoma at TISIDB database.

METTL5 expression presented a strongly positive association with several chemokines of CCL15, CCL16, CCL20, CXCL17, and XCL1 (Fig. 14A). Moreover, METTL5 expression was positively linked with the chemokine receptors of CCR4 and CX3CR1 (Fig. 14B). These findings indicated that METTL5 played a critical function in the immune interaction in HCC.

## Discussion

HCC is a widely recognized prevalent malignant tumor with highly aggressive and fatal biological behavior across the world^10^. An increasing body of evidence revealed that exposure to chronic hepatitis virus infection^11^, persistent alcohol addiction^11^, and host tumor immune microenvironment^12^ were principally responsible for the initiation and development of HCC. Surgical resection is a widely accepted optimal therapy for HCC patients at the earliest stages; however, even after the surgery of a patient, HCC is still accompanied by a high recurrence rate^13^. Although massive advancement was achieved in the aspect of therapeutic strategies in recent years, the clinical survival of HCC patients was far from satisfactory. Thus, promising diagnostic and prognostic biomarkers in HCC are needed to enhance the potential of precision treatments.

Cancer cells are typically characterized by the malignant biological behavior of unrestrained cellular growth^14^. Recent studies reported that cancer cells exhibit the molecular characteristic of misregulated ribosome biogenesis^15^. m6A modification of rRNAs at the sixth adenine site at position 1832 of 18S rRNA (m6A1832) is emerging as a crucial oncogenic signal that facilitates carcinogenesis and rapid progression^16^. METTL5 was widely recognized as the methyltransferase that took charge of specifically catalyzing 18S rRNA m6A1832 modification^17^. A growing body of evidence displayed that the aberrant expression of METTL5 existed in a wide variety of human malignant tumors. A previous study reported that METTL5 is crucial for breast cancer cell growth^7^. Previous researchers clearly proposed that METTL5 expression considerably upregulated in HCC cells, targeted the knockdown of METTL5 suppressed proliferation and invasion abilities of HCC cells, and further induced cancer cell apoptosis in vitro tests^18^. Recent studies emphasized that METTL5 stimulated the proliferation and invasion activity of tumor cells in pancreatic cancer^19^. The study was conducted to evaluate the influence of METTL5 on the tumorigenesis and survival of HCC.

The correlation between METTL5 gene and HCC expressions was investigated using various databases. Our analysis exhibited that METTL5 gene expression was markably elevated in HCC. Furthermore, the METTL5 gene expression gradually increased with the development of the severity of liver cancer. Recent studies revealed that METTL5 functioned as an oncogene that produced a marked effect on the hyperactivation of tumor cell proliferation, migration, and invasion^19^. HCC patients had the worst survival in the presence of enhanced METTL5. Our study revealed that intensified mRNA expression of METTL5 were observed in HCC specimens in comparison with adjacent healthy tissues. METTL5 mRNA in HCC specimens was up-regulated in N1, stage 4, and grade 4 diseases. Our study reported that the overexpression of METTL5 mRNA had a close association with poor OS, PFS, and RFS among HCC patients. Increased METTL5 mRNA expression was strongly linked with unfavorable OS and PFS in stage 3, stage 3 + 4, and AJCC T3 diseases and male HCC patients. METTL5 protein expression was substantially elevated in liver cancer when compared with paired healthy tissues. The overexpression of the METTL5 protein had adverse effects on the prognosis of HCC. The trend of METTL5 expression in the aspects of transcript and protein was substantially in line with that of the gene, that is, METTL5 gene, transcript, and protein have the same influence on the prognosis of HCC. These observations strongly supported that METTL5 might be taken as a potential diagnostic and prognostic indicator for HCC patients. The final results exhibited that the hypermethylation of METTL5 indeed enhanced the expression of METTL5 in HCC samples.

Related functional networks of METTL5 gene are significantly implicated in ribosome signaling, oxidative phosphorylation, spliceosome, proteasome, RNA transport, and mismatch repair, which were consistent with the previous findings that METTL5 alterations engaged in post-transcriptional regulation and ribosome biogenesis^20^. Existing literature reports indicated that METTL5 played an essential role in the promotion of translation initiation^7^, and previous studies revealed that METTL5 participated in the modulation of mismatch repair^21^.

Immune infiltration played a pivotal role in the carcinogenesis and rapid progression of HCC^22^. The infiltration of immune cells was an essential component of the immune microenvironment and contributed to the mediation of tumor proliferation and metastasis^23^. TIMER tools were further adopted to investigate the underlying correlation of immune infiltration and HCC. METTL5 expression showed positive correlation with the infiltration degrees of B cells, CD8+ T cells, CD4+ T cells, macrophages, neutrophil, and dendritic cells in HCC. Accumulative evidence suggested that the interplay between neutrophils and circulating tumor cells could facilitate distant metastasis of tumor cells^24^, and macrophages were proven to enhance tumor cell migration and invasion by promoting the EMT process^25^. Our data revealed that METTL5 expression had an intimate link with the majority of the examined marker genes of B cells, CD8+ T cells, M1 macrophage, neutrophil, dendritic cell, Th1 cells, Th2 cells, and other known immune cells. Moreover, increased METTL5 expression showed a significant correlation with immunostimulators, immunoinhibitors, chemokines, and chemokine receptors. The aforementioned results proved the potential immune function of METTL5 in HCC, implying that its overexpression resulted in HCC by mediating the immune contexture.

Numerous studies identified PD-1 as a principally expressed in CD4+ T cells, and other types of immunocyte and the high abundance of PD-1 contributed to the modulation of immune evasion^26,27^. The interaction of PD-1 and PD-L1 was regarded as a crucial mechanism for evading anti-tumor immunity^28^. CTLA-4 served as the immunoinhibitory molecule universally produced by highly activated Treg cells^29^. PD1/PDL1 and CTLA-4 were indispensable components of immune checkpoint inhibitors; PD1/PDL1 or CTLA-4 checkpoint blockade therapy exhibited potential therapeutic effects on HCC patients^30^. Our results suggested that METTL5 expression had a positive link with PD1 (PDCD1) and CTLA4 in HCC specimens. The aforementioned results reflected that METTL5 might be used as a promising target in antitumor immunotherapy.

Our study suggested that METTL5 expression in HCC specimens had a close link with kinase networks, such as myosin light chain kinase (MLCK), aurora kinase B (AURKB), ribosomal protein S6 kinase A4 (RPS6KA4) functioned on modulating mitosis, cell cycle checkpoint, DNA damage response, cell growth, and cell proliferation^31,32^. AURKB produced marked effects on tumorigenesis and genomic instability^31^. MLCK participates in the regulation of cellular processes for cell adhesion and migration^33^. MLCK was identified as a critical modulator of mitotic division^34^, and the decline in MLCK expression or MLCK activity inhibition highly attenuated the proliferation capacity of different kinds of cancerous cells^35,36^. RPS6KA4 was considered to play the cancer-promoting effects of HCC^32^.

MiRNAs refer to short noncoding RNAs (20–24 nucleotides) that serve as key molecular components of post-transcriptional regulation in controlling gene expressions to facilitate human oncogenic transformation^37^. Our results demonstrated several miRNAs with positive link with METTL5, and accumulative evidence supported that MIR-127 and MIR-423 could act as potential diagnostic and prognostic biomarkers of HCC, respectively^38,39^. The hyperactivation of MIR-127 regulates NF-κB signaling to inhibit HCC cell proliferation^40^. miR-423 was discovered to contribute to HCC progression by modulating the BP of cell growth and cell cycle^41^. MIR-510 also participated in tumor development^42^. The aforementioned results implied that the dysregulation of these miRNAs led to the initiation and progression of HCC.

Several limitations existed in our study. Firstly, most of our data were principally extracted on the basis of online platform databases. Secondly, there were few data from public databases, which were utilized to analyze the relationship between chronic liver diseases and METTL5 expression, so we couldn’t further investigated whether METTL5 expression contributed to HCC occurrence and progression by affecting the process of chronic liver diseases. Hence, further experimental validation must be conducted in subsequent studies. Finally, the assessment of “low” and “high” METTL5 expression was not elaborated in these online public databases, a more precise analysis should be conducted if a detailed and accurate cut-off level of METTL5 expression is set.

## Conclusions

METTL5 expressions were markedly upregulated among HCC patients. Increased METTL5 expression had detrimental effects on the survival of HCC patients. METTL5 overexpression affects the carcinogenic effect by taking part in the regulation of tumor immunity. Thus, targeting METTL5 might shed new light on the improvement of immunotherapy effectiveness.

## Materials and methods

### GEPIA analysis

GEPIA (http://gepia.cancer-pku.cn/) obtained the gene expression and clinical data of malignancy samples from the TCGA databases^43^. We used GEPIA to examine the METTL5 gene expression among multiple types of malignancies. We also assessed the effects of METTL5 expression on the clinical outcomes of liver HCC patients through the “Survival” module. Statistical significance was represented by a cutoff value of p < 0.05.

### UALCAN

UALCAN (http://ualcan.path.uab.edu/) provided the extensive expression information of mRNA, promoter methylation, and protein and survival parameters of multiple malignancy type samples from the TCGA database^44^. The original expression data of the METTL5 gene, transcript, protein, and methylation in cancer were compared with healthy samples, and the prognostic effect of METTL5 was evaluated through the UALCAN database. Statistical significance was set as a p-value < 0.05.

### Kaplan–Meier (KM) Plotter

The KM plotter (http://kmplot.com/analysis/)^45^ was applied to estimate the effects of METTL5 genes on the survival outcomes among liver cancer patients. Statistical significance was set as a p-value < 0.05.

### HPA

HPA databases^46^ were used to illustrate the effect of METTL5 protein on the survival outcomes of liver cancer patients. A p-value < 0.05 was considered statistically significant.

### cBioPortal

cBioPortal (http://www.cbioportal.org/)^47^ was utilized to visualize the frequency of the genetic alteration of METTL5 in the HCC samples via OncoPrint module.

### LinkedOmics

Various analyses of functional enrichment, kinase-target enrichment, and miRNA-target enrichment were conducted by analyzing cancer-associated datasets through the LinkedOmics websites (http://linkedomics.org/admin.php)^48^. The meta p-value was analyzed by utilizing “RankCriteria”, the “Minimum Number of Genes Size” was set as 3 and the “Simulations” was set as 500.

### Metascape

Metascape (http://metascape.org/gp/index.html) was comprehensively used to generate interactive METTL5 genes^49^.

### GeneMANIA

Protein–protein interaction (PPI) networks were constructed through the publicly available platform of GeneMANIA (http://genemania.org/)^50^. Interactive METTL5 genes derived from the Metascape dababase were inputted into the GeneMANIA for the assessment of gene functions.

### TIMER

TIMER (https://cistrome.shinyapps.io/timer/) was applied to systematically assess the correlation of METTL5 with immune cell infiltration in various malignancies and evaluate the impact of immune infiltration on survival outcomes^51^. The relationship between METTL5 expressions and the levels of immune cell infiltration in LIHC were assessed by utilizing the “Gene” module. The correlation between the copy number alterations of METTL5 and the levels of immune cell infiltration through the “SCNA” module.

### TISIDB

TISIDB (http://cis.hku.hk/TISIDB/index.php)^52^ was applied to explore the interaction between human tumors and immunoregulatory factor for cancer immunology research.

### Ethics approval and consent to participate

Ethics approval from the Institutional Review Board and written informed consent from eligible patients were not required because the data are publicly accessible and patient’s private information was deleted.

## Data Availability

Publicly available datasets were analyzed during the present study. This data can be found here: http://gepia.cancer-pku.cn/.
